# Polyp Segmentation with Fully Convolutional Deep Neural Networks—Extended Evaluation Study †

**DOI:** 10.3390/jimaging6070069

**Published:** 2020-07-13

**Authors:** Yunbo Guo, Jorge Bernal, Bogdan J. Matuszewski

**Affiliations:** 1 Computer Vision and Machine Learning (CVML) Group, School of Engineering, University of Central Lancashire, Preston PR1 2HE, UK; bmatuszewski1@uclan.ac.uk; 2 Image Sequence Evaluation laboratory, Computer Vision Center and Computer Science Department, Universitat Autònoma de Barcelona, 08193 Bellaterra, Barcelona, Spain; jorge.bernal@uab.cat

**Keywords:** fully convolutional dilation neural networks, polyp segmentation, data augmentation, cross-validation, ablation tests

## Abstract

Analysis of colonoscopy images plays a significant role in early detection of colorectal cancer. Automated tissue segmentation can be useful for two of the most relevant clinical target applications—lesion detection and classification, thereby providing important means to make both processes more accurate and robust. To automate video colonoscopy analysis, computer vision and machine learning methods have been utilized and shown to enhance polyp detectability and segmentation objectivity. This paper describes a polyp segmentation algorithm, developed based on fully convolutional network models, that was originally developed for the Endoscopic Vision Gastrointestinal Image Analysis (GIANA) polyp segmentation challenges. The key contribution of the paper is an extended evaluation of the proposed architecture, by comparing it against established image segmentation benchmarks utilizing several metrics with cross-validation on the GIANA training dataset. Different experiments are described, including examination of various network configurations, values of design parameters, data augmentation approaches, and polyp characteristics. The reported results demonstrate the significance of the data augmentation, and careful selection of the method’s design parameters. The proposed method delivers state-of-the-art results with near real-time performance. The described solution was instrumental in securing the top spot for the polyp segmentation sub-challenge at the 2017 GIANA challenge and second place for the standard image resolution segmentation task at the 2018 GIANA challenge.

## 1. Introduction

Colorectal cancer (CRC) is one of the major causes of cancer incidence and death worldwide; e.g., in the United States, it is the third largest cause of cancer deaths, whereas in Europe, it is the second largest with 243,000 deaths in 2018. Globally there were 1,096,601 new cases and 551,269 deaths in 2018 [1]. Colon cancer’s five-year survival rate depends strongly on an early detection—decreasing from 95%, when detected early, to only 35% when detected in the later stages [2,3], hence the importance of colon screening. It is commonly accepted that the majority of CRCs evolve from precursor adenomatous polyps [4]. Typically, a colonoscopy screening is proposed to detect polyps before any malignant transformation or at an early cancer stage. Optical colonoscopy is the gold standard for colon screening due to its ability to discover and treat the lesions during the same procedure. However, colonoscopy screening has significant limitations. Various recent studies [5,6] have reported that between 17% and 28% of colon polyps are missed during routine colonoscopy screening procedures, with about 39% of patients having at least one polyp missed. It has also been estimated that an improvement of polyp detection rate by 1% reduces the risk of CRC by 3% [7]. It is therefore essential to improve analysis of colonoscopy images to ensure more robust and accurate polyp characterization, as it can reduce the risk of colorectal cancer and healthcare costs. With new advanced machine learning (ML) methodologies, it is conceivable to significantly increase the robustness and effectiveness of CRC screening.

Clinicians have identified lesion detection and classification as the two most relevant tasks for which intelligent systems can play key roles in improving the effectiveness of the CRC screening procedures [8]. Lesion segmentation can be used to determine whether there is a polyp-like structure in the image, assist in polyp detection, and accurately delineate the polyp region, which helps with lesion histology prediction [9]. This paper provides a comprehensive study showing how polyp segmentation can be effectively addressed by using deep learning methodology.

**Figure 1:**
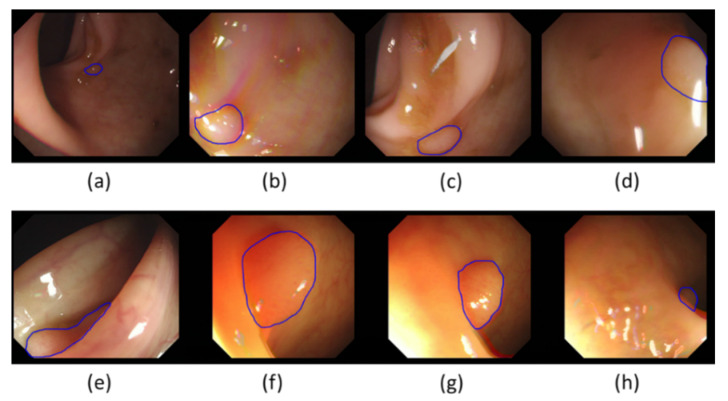
A sample of typical polyps from the GIANA SD (CVC-ColonDB) training dataset: (**a**,**h**) Small size. (**b**) Blur. (**c**) Intestinal content. (**d**) Specular highlights/defocused. (**e**) Occlusion. (**f**) Large size. (**g**) Overexposed areas. (**a**,**e**,**h**) Luminal region.

Segmentation is an essential enabling technology for medical image analysis with a great variety of methods [10,11,12,13,14,15,16]. Analysis of colonoscopy images, including polyp segmentation, is a challenging problem because of intrinsic image appearance and polyp morphology variabilities. Polyp characteristics change at different development stages, with evolving polyp shape, size, and appearance (see Figure 1). Initially, colorectal polyps are generally small, with no strongly distinctive features. Therefore, at this stage they could be easily mistaken for other intestinal structures, such as wrinkles and folds. Later, when they evolve, they often get bigger and their morphology changes, with the features becoming more distinctive. Polyps’ appearance in colonoscopy images is also strongly dependent on the imaging devices used and illumination conditions. Illumination can produce substantial image changes with varying local overexposure highlights, specular reflections, and low contrast regions. Polyps may look very different even from slightly altered camera positions and may not have visible transitions between themselves and their surrounding tissues. Furthermore, various other artefacts, such as defocus, motion blur, intestinal content, and possibly surgical instruments present in the camera view, can inevitably lead to segmentation errors. 

With new, advanced machine learning methodologies, including the so-called deep learning, it seems conceivable to significantly increase the robustness and effectiveness of colorectal cancer screening, and improve the segmentation accuracy, lesion detectability, and the accuracy of the histological characterization. In recent years there have been several efforts to apply advanced machine learning tools for automatic polyp segmentation. This work has been inspired by the limitations of these previously proposed methods. This paper is focused on the evaluation of a novel fully convolutional neural network (FCN) specifically developed for this challenging segmentation task. The proposed FCN system outputs polyp incidence probability maps. The final segmentation result was obtained either by a simple thresholding of these maps, or a hybrid level-set [17,18] was used to smooth the polyp contour and eliminate small, noisy network responses.

The paper is an extended version of the paper presented at the 2019 Medical Image Understanding and Analysis (MIUA) conference [19]. Compared to that conference paper, this paper extends the analysis by proving more in-depth evaluation, including added justification for the network architecture, test-time data augmentation, analysis of the cross-validation, results visualization, processing-time, and comparisons with other segmentation methods evaluated on equivalent segmentation problem.

## 2. Related Work

Segmentation has an important role to play in colonoscopy image analysis. This becomes clear even when considering how different terminology is used to describe similar processes and objectives. Indeed, many existing polyp detection and localization methods can already be interpreted as segmentation as they provide heat maps and different levels of polyp boundary approximations. Equally, segmentation tools can be used to provide polyp detection and localization functionality and they can be of great help when delimiting the region to be analyzed for a posterior automatic histology prediction process. Polyp segmentation methods can generally be divided into two categories, those based on shape and those based on texture, with machine learning methods becoming more popular in both categories.

The earliest approaches in the shape-based segmentation category used predefined polyp shape models, required manual contour initialization heavily depending on the presence of complete polyp contours, and were less effective for atypical polyps. Hwang et al. [20] used ellipse fitting techniques based on image curvature, edge distance, and intensity values for polyp detection. Gross et al. [21] used the Canny edge detector to process prior-filtered images, identifying the relevant edges using a template matching technique for polyp segmentation. Breier et al. [22,23] investigated applications of active contours for finding polyp outline.

The shortcomings of these early methods led to the development of robust edge detectors. The “depth of valley” concept was introduced by Bernal et al. [24] and further improved in [25,26,27]. This allowed one to detect more general polyp shapes, and then segment the polyp through evaluating the relationship between the pixels and detected contour. Later work by Tajbakhsh et al. [28] used edge classification, utilizing the random forest classifier and a voting scheme producing polyp localization heat maps, and was then improved via the use of sub-classifiers [29,30].

Polyp segmentation methods within the texture category typically operated on a sliding window. Karkanis et al. [31] combined gray-level co-occurrence matrix and wavelets. Using the same database and classifier, Iakovidis et al. [32] proposed a method that provided the best results in terms of area under the curve metric.

Advances in deep learning techniques have recently allowed the gradual replacement of hand-crafted feature descriptors by convolutional neural networks (CNN) [33,34]. Ribeiro et al. [35] looked at a CNN with a sliding window approach and found that it performed better at polyp classification than the state-of-art hand-crafted feature methods. However, sliding window techniques are inefficient and find it difficult to use image contextual information. Consequently, an improvement has been suggested in the form of fully convolutional networks (FCN) [36,37], which can be trained end-to-end and output complete segmentation results, without the need for any post-processing. As shown by Vázquez et al. [38], even a retrained off-the-shelf FCN produced competitive polyp segmentation results. Zhang et al. [39] showed that the same architecture can be improved by adding a random forest to decrease false positives. One of the most popular architectures for biomedical image segmentation, the U-net [37], was used by Li et al. [40] to encourage smooth contours in polyp segmentation.

The existence of a relationship between the size of the CNN receptive field and the quality of segmentation results has prompted the introduction of a new layer, the dilation convolution, in order to control the CNN receptive field in a more efficient way [41]. Chen et al. [42] utilized this to develop a new architecture called atrous spatial pyramid pooling (ASPP). The ASPP module consists of multiple parallel convolutional layers with different dilations to facilitate learning of multi-scale features.

There is an increasing trend of colonoscopy image analysis becoming more automated and integrated, making use of more machine learning techniques. Polyp segmentation can be seen as a semantic instance segmentation problem, and therefore many of the techniques and improvements developed for a generic semantic segmentation can possibly be adopted for polyp segmentation. There is a clear trend of deep feature learning and the end-to-end architectures gradually replacing hand-crafted and deep features operating on a sliding window. All of this allows for significant improvements, creating more efficient and accurate methods for polyp segmentation.

## 3. Method

**Figure 2:**
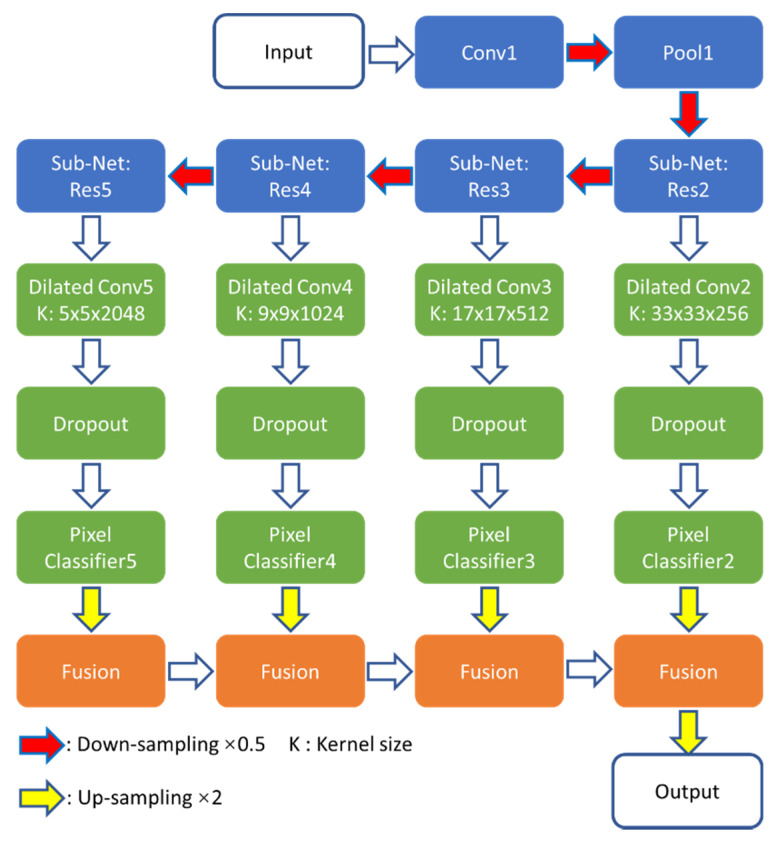
Dilated ResFCN network architecture with feature extraction, classification, and fusion sub-networks shown respectively in blue, green, and orange.

The Dilated ResFCN polyp segmentation network investigated in this paper, previously introduced in [19,43], is shown in Figure 2. Its design was inspired by the FCN [36], DeepLab [42], and Global Convolutional [44] networks. The proposed solution consists of feature extraction, multi-resolution classification, and fusion sub-networks. 

The ResNet-50 model [45] has been selected as the feature extraction sub-network because, as shown later in this section, it provides a reasonable balance between network capacity and required resources for the polyp segmentation problem. The ResNet-50 can be partitioned into five constituent parts: Res1–Res5 (see Table 1 in [45]). Res1 includes the first convolutional (Conv1) and pooling (Pool1) layers. Res2–Res5 incorporate sub-networks, having respectively 9, 12, 18, and 9 convolutional layers with 256, 512, 1024, and 2048 feature maps. Each of these subnets works on gradually reduced maps, down sampled with stride two when moving from right to left (Figure 2). The sizes of these feature maps are 62 × 72, 31 × 36, 16 × 18, and 8 × 9 respectively.

The classification sub-network encompasses four multi-resolution parallel paths connected to the outputs from Res2–Res5 of the feature extraction sub-network. Each parallel path consists of dilation and 1 × 1 (denoted as “Pixel Classifier”) convolution layers to, respectively, assemble image information at different spatial ranges and perform the pixel-level classification at different resolution levels. Furthermore, a dropout layer [46] is included to improve the network training performance. The results section shows that the key to the success of the proposed architecture is the use of dilated kernels, as they increase the receptive field without increasing network information capacity or computational complexity. With the large receptive field, the network can extract long range dependencies, incorporating image contextual information into pixelwise decisions. The process of selecting suitable dilation rates in each of the parallel paths of the classification sub-network is explained in some detail later in this section. The essential part of that process is to match the receptive field to the statistics of polyp size. Based on the available training data, the 5 × 5 kernel was selected for the lowest resolution path, which is connected to Res5 (see Figure 2). This kernel corresponds to a dilation rate of 2 and it can adequately represent 91% of all polyps in the training dataset. The regions of dilation convolutions should be overlapping, and therefore the dilation rates increase with resolution. The dilation rates for sub-nets connected to Res4–Res2 are 4, 8, and 16 and the corresponding kernel sizes are 9, 17, and 33. The fusion sub-network corresponds to the deconvolution layers of the FCN model. In the proposed architecture, a bilinear interpolation is used to up-sample results from each multiresolution classification path to match the image sizes, and the fusion is completed by simply adding such aligned corresponding sub-network responses.

**Figure 3:**
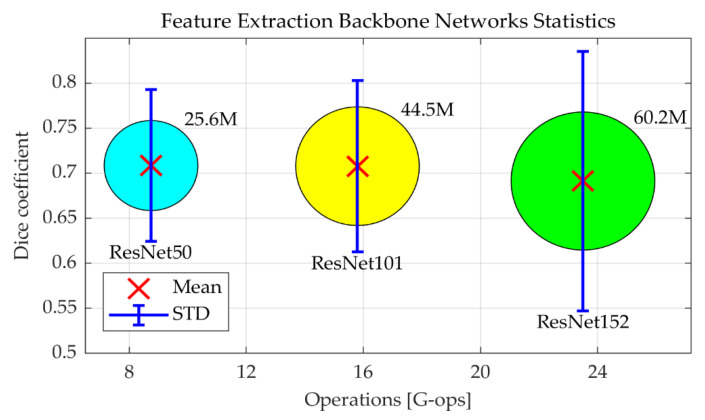
Chart comparing performance of three tested CNNs, explaining the selection of the backbone feature extraction network in the Dilated ResFCN architecture. Number of operations corresponds to a single forward pass of the backbone network. The number of parameters for each network is represented by the corresponding circle [47]. The values shown as red crosses were obtained using Dilated ResFCN architecture with ResNet50, ResNet101, and ResNet152 as feature extraction backbone networks (shown as blue in Figure 2), with the corresponding standard deviation estimates represented by the blue vertical bars.

As explained above, ResNet-50 is selected as the backbone network for image feature extraction, as it has been previously shown to provide a good compromise between accuracy, required memory footprint, and computational requirements [47]. However, the question is whether deeper networks would help to improve the segmentation performance. To test this, the ResNet-50 in the Dilated ResFCN architecture, shown in blue in Figure 2, was replaced by ResNet-101 and ResNet-152. Figure 3 shows, as red crosses, the corresponding mean Dice coefficients, and the number of operations required for each given backbone network. The corresponding Dice coefficient standard deviation (STD) is represented by the vertical bars. The area of the circles represents the number of parameters for the corresponding network. ResNet-152 is the worst one, with the smallest mean Dice coefficient; and the largest standard deviation, number of operations, and required number of parameters. ResNet-101 and ResNet-50 have similar mean values, but ResNet-101 has a larger standard deviation and larger numbers of parameters and operations. Therefore, the segmentation performance gets worse when a deeper backbone network is used. This can possibly be explained by the fact that, for the given polyp segmentation problem, ResNet-101 and ResNet-152 provide too much information capacity, leading to overfitting.

**Figure 4:**
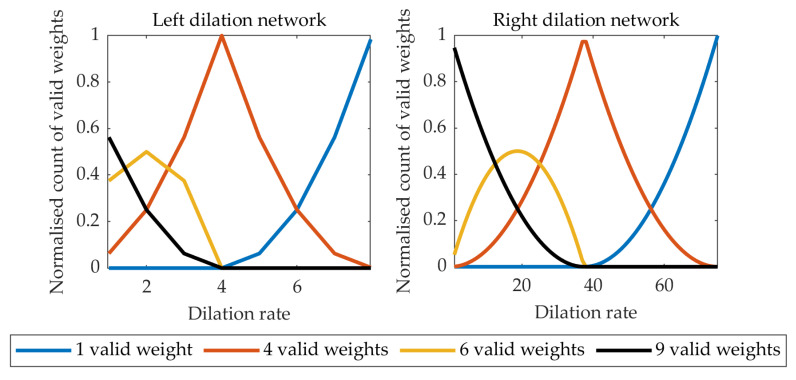
The count of valid weights in the dilation kernel for the left and right classification sub-networks in Figure 2.

Following the methodology described in [48], the numbers of active kernel weights in the left and right paths of the classification subnetwork are shown in Figure 4. It can be seen that with the dilation rate too high, the 3 × 3 kernel is effectively reduced to a 1 × 1 kernel. On the other hand, too small a dilation rate leads to a small receptive field negatively affecting the performance of the network. The selected dilation rates of 2 and 16, for the “left” and “right” networks respectively, provide a compromise with a sufficient number of kernels having 4–9 valid weights.

The Dilated ResFCN feature extraction module (ResNet-50) was initialized with the weights trained on the ImageNet database. The remaining convolutional and up-sampling sub-networks were initialized by Xavier [49] and bilinear interpolation weights respectively. Subsequently, the whole network was trained on the polyp training dataset. Dilated ResFCN was trained using the Adam algorithm [50] using the Caffe framework. They were trained with thirty epochs and an initial learning rate of 10^−4^, which was reduced by a factor of 0.1 after every 10 epochs.

## 4. Data

The proposed polyp segmentation method has been developed and evaluated on the data from the 2017 Endoscopic Vision GIANA Polyp Segmentation Challenge [51]. The data includes both standard definition (SD) and high definition (HD) polyp images. 

The SD set consists of the publicly available CVC-ColonDB [24,52] used for training and the CVC-ClinicDB [26,53,54] used for testing. The CVC-ColonDB contains 300 polyp images acquired from 15 video sequences, all of them showing different polyps. Each image has a resolution of 500 by 574 pixels. On average, 20 images were selected from each sequence, warranting the maximum variability between images of the same polyp. Each image has a corresponding pixel-wise mask, annotated by an expert, to create the associated ground truth data. The CVC-ClinicDB contains 612 polyp images selected from 31 colonoscopy video sequences, each showing a different polyp. As in the case of the CVC-ColonDB, an average of 20 images, each of 288 by 384 pixels, were selected from each video, with the aim of having as many different polyp views as possible. In this case, several experts contributed to the definition of ground truth data. A single expert generated the pixel-wise masks representing the polyp regions and the remining experts contributed to the definition of the clinical metadata describing each polyp characteristics.

The GIANA HD dataset [51] is a collection of high-definition white light colonoscopy images assembled as part of the effort to develop and validate methods for automatic polyp histology prediction [9]. These data of 1080 by 1920 pixel images is divided into two subsets. The fist subset with 56 images was used for training, and the second subset with 108 images was used for testing. For the ground truth, an expert generated pixel-wise binary masks using GTCreator [55], which were subsequently reviewed by a panel of experts. 

The results reported in this paper are based on a cross-validation approach using the training dataset only. Selected results obtained on the SD test dataset were reported in [43]. 

### Data Augmentation

The performance of the CNN-based methods relies heavily on the size of training data used. Clearly, the set of training images is very limited in this case, at least from the perspective of a typical training set used in a context of deep learning. Moreover, some polyp types are not represented in the database, and for some others there are just a few exemplar images available. Therefore, it is necessary to enlarge the training set via data augmentation. Data augmentation is designed to provide more polyp images for CNN training. Although this method cannot generate new polyp types, it can provide additional data samples based on modelling different image acquisition conditions, e.g., illumination, camera position, and colon deformations.

**Figure 5:**
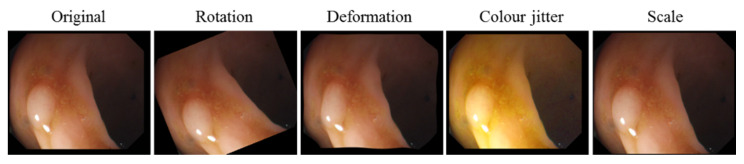
An example of an standard definition (SD) image augmentation using rotation, local deformation, color jitter, and scale.

All training images are rescaled to a common image size (250 by 287 pixels) in such a way that image aspect ratio is preserved. This operation includes random cropping equivalent to image translation augmentation. Subsequently, all images are augmented using four transformations. Specifically, each image is: (i) rotated with the rotation angle randomly selected from a range of 0°–360°, (ii) scaled with the scale factor randomly selected between 0.8 and 1.2, (iii) deformed using a thin plate spline (TPS) model with a fixed 10 × 10 grid and a random displacement of the each grid point with a maximum displacement of 4 pixels, and (iv) color adjusted, using color jitter, with the hue, saturation, and value randomly changed, with the new values drawn from the distributions derived from the original training images [56]. After augmentation, the training dataset consists of 19,170 images in total. A representative sample of the augmented images is shown in Figure 5.

For the purpose of validation, the original training images were divided into four V1–V4 cross-validation subsets with 56, 96, 97, and 106 images respectively. Following augmentation, the corresponding sets had 4784, 4832, 4821, and 4733 images for training. Following the standard 4-fold validation scheme, any three of those subsets were used for training (after image augmentation) and the remaining subset (without augmentation) for validation. Frames extracted from the same video are always in the same validation sub-set; i.e., they are not used for training and validation at the same time.

**Figure 6:**
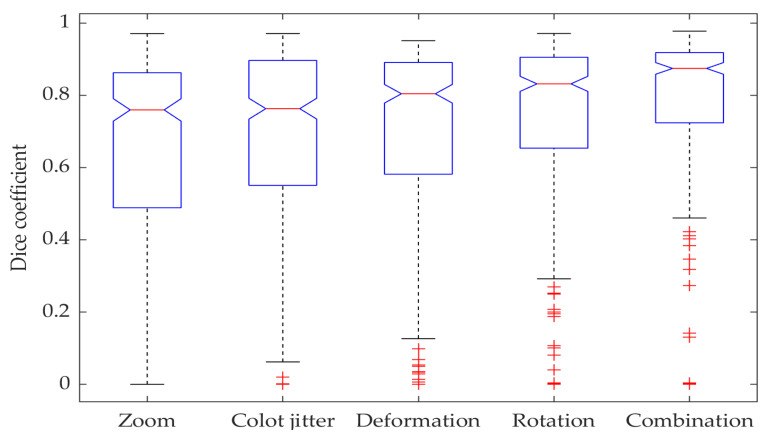
Box plot showing Dice coefficient results for data augmentation ablation tests with dilated ResFCN network.

Figure 6 demonstrates the impacts different augmentation techniques have on network performance. The ablation tests show that the combination of different augmentation techniques significantly improves the segmentation results when compared to any standalone augmentation method, with the real combined method median being better than any other individual augmentation median with a 95% confidence level.

## 5. Results

### 5.1. Evaluation Metrics

Evaluation of image segmentation quality is an important task, particularly for biomedical applications. Suitable evaluation measures should reflect segmented object properties relevant to the specific application [57]; e.g., when the precise location of the organ/tissue boundaries is of concern. the spatial distance measures, such as Hausdorff distance or boundary Jaccard index [58], should be used. When overall organ/tissue location is of importance, the overlap based measures are often used (e.g., Dice coefficient, Jaccard index, precision, and recall), whereas when comparative segmentation quality is needed without access to the ground truth, pair-counting-based methods are used (e.g., Rand index or adjusted Rand index). The GIANA challenges [51] have become a benchmark for the assessment of colonoscopy polyp analysis, and therefore it has been decided to use the measures adopted by the GIANA challenges.

The Dice coefficient (also known as F1 score), precision, recall, and the Hausdorff distance are used, for each detected polyp, to compare the similarity between the binary segmentation results and the ground truth. For the results reported here, a simple thresholding operation on the networks output was used, followed by the morphological opening and hole filling operations to create binary networks’ response.

Precision and recall are standard measures used in a context of binary classification:

(1) $$Precision=\frac{TP}{TP+FP}\;Recall=\frac{TP}{TP+FN}$$

where *TP*, *FP*, and *FN* denote, respectively, true positive, false positive, and false negative. Precision and recall can be used to quantify the over-segmentation and under-segmentation. The Dice coefficient is one of standard evaluation measures used in image segmentation and is defined as:

(2) $$Dice=\frac{2\times TP}{2\times TP+FP+FN}$$

It should be noted that the other popular overlap-based measure, the *Jaccard* index:

(3) $$Jaccard=\frac{TP}{TP+FP+FN}$$

can be expressed as function of the Dice coefficient:

(4) $$Jaccard=\frac{Dice}{2-Dice}$$

Therefore, the evaluation reported here is based on the Dice coefficient only.

The Hausdorff distance provides the means to quantify similarities between the boundaries *G* and *S* of two objects, with zero indicating that the object contours completely overlap. It is defined as:

(5) $$H\left(G,S\right)=max\left\{sup_{x\in G}\;inf_{y\in S}\;d\left(x,y\right),sup_{x\in S}\;inf_{y\in G}\;d\left(x,y\right)\right\}$$

where d(x,y) denotes the distance between points x and y. It should be noted that the Hausdorff distance is sensitive to outliers and is not bounded. For these reasons it has been argued that shape measures such as the boundary Jaccard index, which is both robust to outliers and bounded, should be used instead. However, due to the above-mentioned compatibility with the results reported for the GIANA challenges, the Hausdorff distance is also used in this paper.

### 5.2. Test Time Data Augmentation

**Figure 7:**
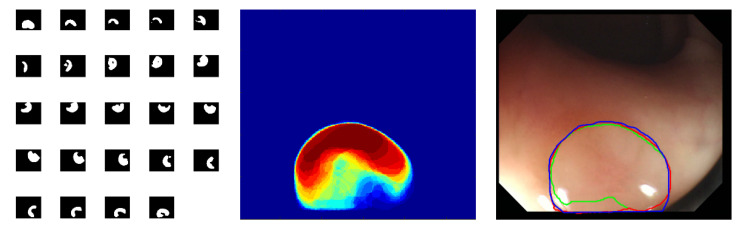
Graphical representation of the test time data augmentation; the images on the left show the network binary outputs for each rotation augmented image; the image in the middle shows the results of averaging of these binary images after transferring them into the original image reference frame; the image on the right shows final segmentation results, superimposed on the original image, with three contours showing ground truth (in blue), results without data augmentation (in green), and with the data augmentation (in red).

The proposed deep segmentation networks are not rotation invariant, so it is possible to perform additional rotation data augmentation in the prediction mode to tweak their segmentation accuracy. The adopted implementation of the test time augmentation uses 24 images obtained from the original input image, which is rotated in 15 degrees intervals. These 24 augmented images are subsequently presented to the network input and the corresponding output images are averaged after being converted to the original image reference frame. The whole procedure is graphically represented in Figure 7. Although it is possible to implement other types of the test time augmentation, it should be recognized that any such augmentation will increase computation time, and therefore a trade-off is required between quality of segmentation and computation time. For the problem considered in this work, it has been concluded that the rotation-only augmentation approach provides such a trade-off. 

**Figure 8:**
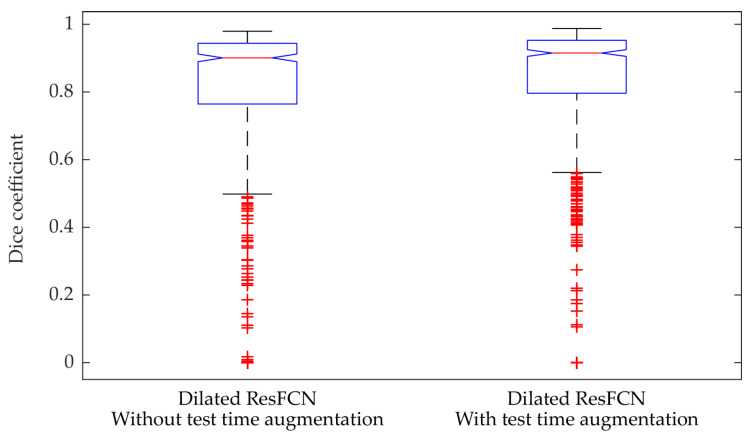
Box plot of the Dice coefficient obtained for the Dialted ResFCN without and with the test time augmentation.

The proposed test time augmentation better utilizes the generalization capabilities of the network. The quantitative comparison of the segmentation results for FCN8s, ResFCN, and Dilated ResFCN with and without data augmentation is shown in Figure 8. For the Dilated ResFCN, the test time data augmentation increases the median, from 0.90 without to 0.92 with the test time augmentation. The distribution of the results with test time augmentation is also more compact.

### 5.3. Results

This section reports on several experiments performed to evaluate the proposed methods and compare their characteristics. The two reference network architectures FCN8s [36] and ResFCN have been selected as benchmarks for comparative analysis of the proposed methods. Whereas FCN8s is a well-known fully convolutional network, the ResFCN is a simplified version of the network from Figure 2, with the dilation kernels removed from the parallel classification paths. The ResFCN is included to verify the significance of the dilated kernels in the Dilated ResFCN model.

Table 1 reports mean Dice coefficients computed on all tested networks on four validation subsets described in Section 4. The last two table columns list the Dice coefficient and standard deviations values averaged over all four validation subsets. The Dilated ResFCN is the best (indicated by bold typeface) on all validation subsets, demonstrating consistent performance with respect to changes in the training data.

**Figure 9:**
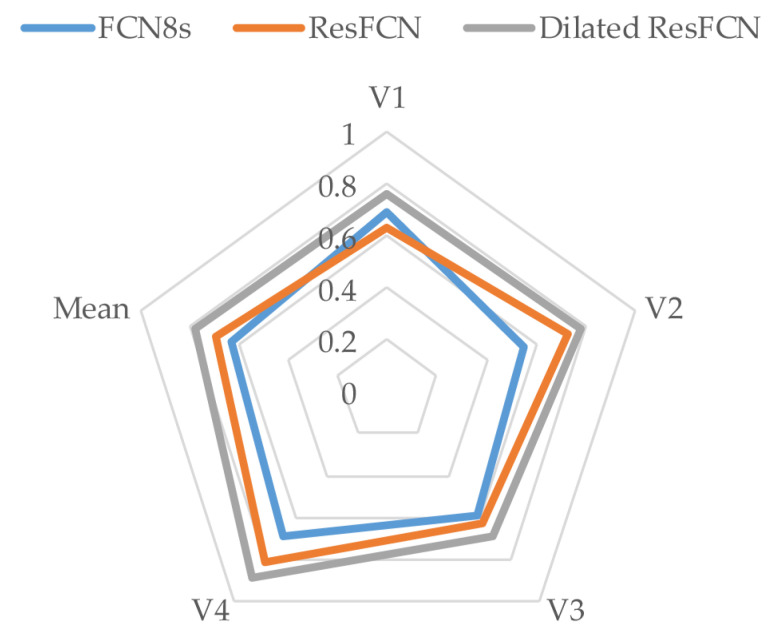
Graphical representation of Dice coefficient results for different segmentation methods overall and different validation subsets. The more outwardly located the contour, the better the results.

The results from Table 1, are represented in a graphical form in Figure 9. It is evident that, on the available data, the Dilated ResFCN has the best performance with the contour representing that method encompassing contours representing other methods.

**Figure 10:**
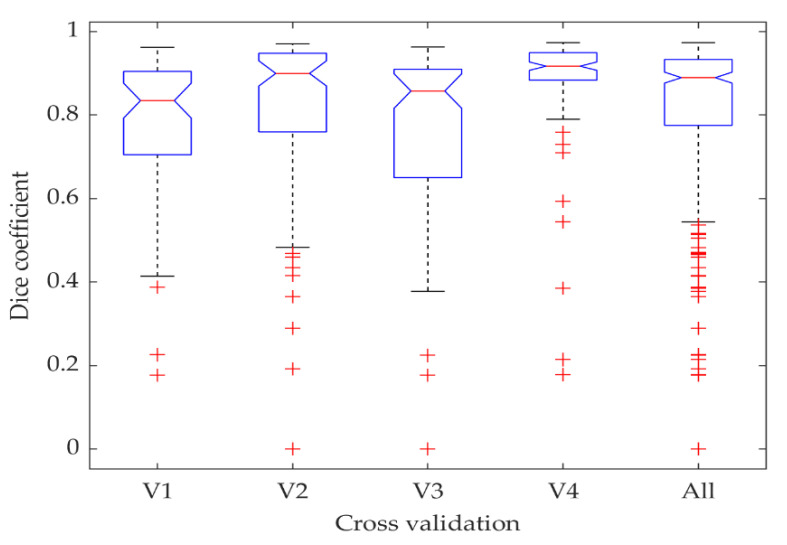
Statistics of the Dice coefficient computed for different cross-validation folds visualized using boxplots.

The distribution of the Dilated ResFCN results as a function of the cross-validation folds is shown in Figure 10. The results obtained on the fourth and third folds are respectively the best and worst. A closer examination of these folds reveals that images in the fourth fold are mostly showing larger polyps, whereas images in third fold are mostly depicting small polyps.

**Figure 11:**
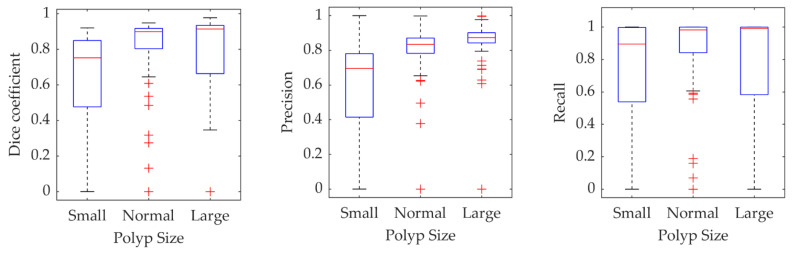
Statistics for different measures on the validation results obtained for the Dilated ResFCN network grouped as a function of the poly size.

To further investigate the performance of the proposed method as a function of the polyp size, Figure 11 shows the boxplot representing Dice coefficient, precision, and recall as functions of the polyp size. The “Small” and “Large” polyps are defined as being smaller than the 25th and larger than the 75th percentile of the polyp sizes in the training dataset. The remaining polyps are denoted as “Normal.” The results demonstrate that the small polyps are hardest to segment. However, it should be said that the measures used are biased towards larger polyps as a relatively small (in pixels) over and under-segmentation for small polyps would lead to more significant deterioration of the measure.

**Figure 12:**
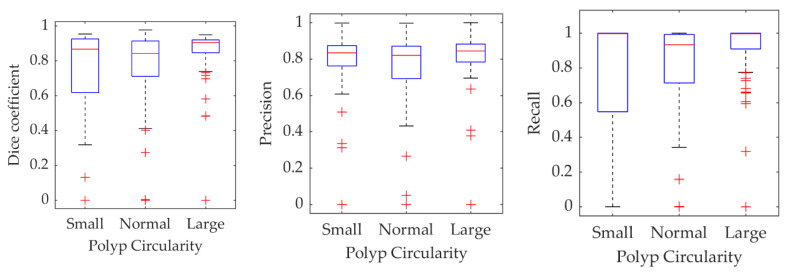
Statistics for different measures on the validation results obtained for the Dilated ResFCN network grouped as a function of the polyp circularity.

To check whether the segmentation results depend on polyp’s shape, a circularity of the polyp ground truth is used as a proxy for shape. It is calculated as a scaled ratio of a polyp area and square of its perimeter, so the values are between zero and one. A perfectly circular shape has a circularity of one, and smaller values correspond to more irregular shapes. As in Figure 11, statistics of the three metrics have been calculated for polyps grouped into three sub-sets with small, normal, and large circularity. The small and large sub-sets were constructed from polyps for which ground truth circularity was smaller than the 25th and larger than the 75th percentile of the training data polyp population circularity. The normal sub-group is constituted from the remining polyps. The results are shown in Figure 12. Although there is some variability of the measures’ statistics in each subgroup, these are not as distinctive as in case of the polyp size.

Table 2 lists the median and mean results for all three tested methods and all four evaluated metrics. As can be seen from that table, the Dilated ResFCN method achieved the best results for all four metrics. It got the highest values for Dice coefficient, precision, and recall, and the smallest value for the Hausdorff distance, demonstrating the stability of the proposed method.

**Figure 13:**
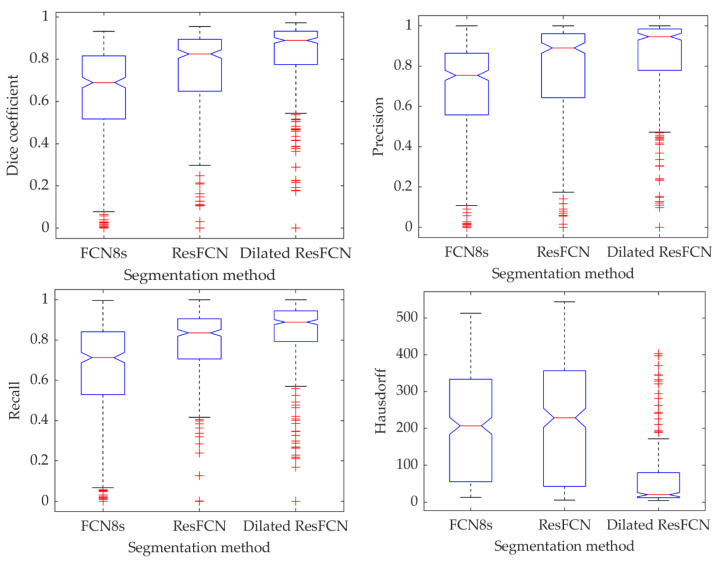
The summative polyp segmentation results for different methods and evaluation measures.

Figure 13 demonstrates the results’ statistics for all the methods and all the measures using box plots, with the median represented by the central red line, the 25th and 75th percentiles represented by the bottom and top of each box, and the outliers shown as the red points. It can be concluded that the proposed methods achieve better results than the benchmark methods. For all the measures, the true medians for the Dilated ResFCN are better, with 95% confidence, than the other methods. The significantly smaller Hausdorff distance measure obtained for the Dilated ResFCN indicates a better stability of that method with boundaries of segmented polyps better fitting the ground truth data. 

**Figure 14:**
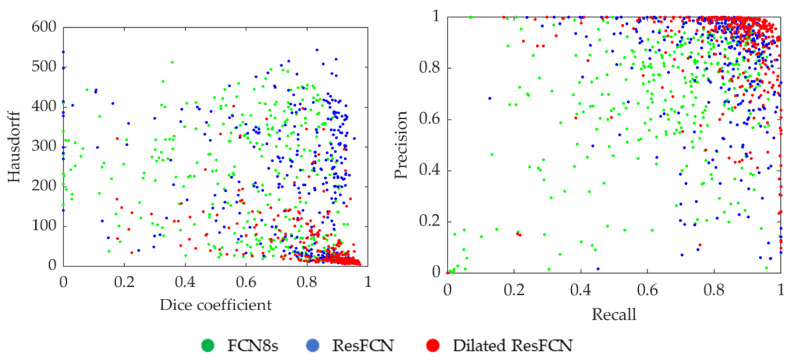
Distribution of the results when using different segmentation methods, where each point corresponds to one of the validation images.

The distribution of segmentation results for all the data points in the Dice coefficient–Hausdorff distance and recall–precision spaces are shown in Figure 14. Each point represents an image, with the color corresponding to the specific segmentation method. It can be seen that the Dilated ResFCN outperforms all other methods, with the majority of corresponding points more tightly grouped together in the bottom right corner of the Dice coefficient–Hausdorff distance space and the top right corner of the recall–precision space. However, even for this method there are some outliers, which seem to be uniformly distributed in both spaces. It could be therefore concluded that the Dilated ResFCN is not biased towards under or over-segmentation. The ResFCN, although better than FCN8s with the majority of points within upper ranges of the Dice coefficient, seems to be poor in correctly detecting polyp boundaries, with a cluster of points on the left-hand side of the Hausdorff distance-Dice coefficient plot.

**Figure 15:**
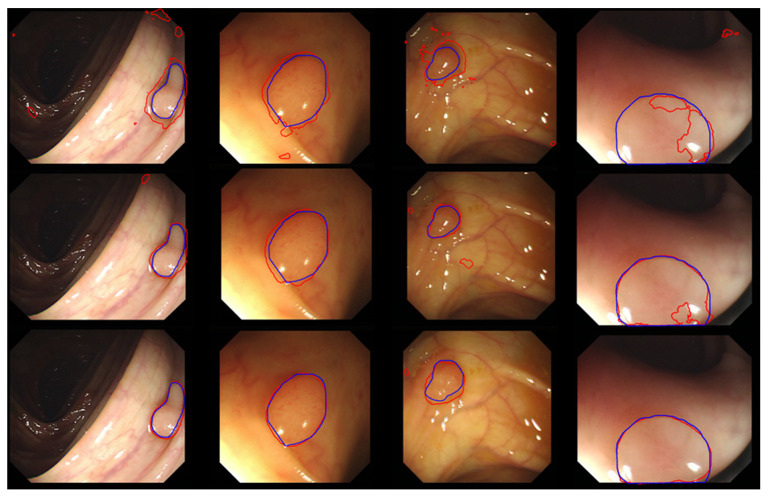
A sample of typical results for the FCN8s (the first row), ResFCN (the second row), and Dilated ResFCN (the last row) with the ground truth represented by the blue contour and the segmentation by the red contour.

Typical segmentation results obtained using the FCN8, ResFCN, and Dilated ResNet networks are shown in Figure 15, with the blue and red contousr representing, respectively, ground truth and the segmentation results.

Mean segmentation processing times for a single image with CPU and GPU implementations are shown in Table 3. When implemented on the GPU all the methods have comparable processing times, enabling near real-time performance. It is expected that further code optimization could provide real-time processing (approximately 30 frames/s).

### 5.4. Comparative Analysis

Section 2 reviews some of the most important polyp segmentation methods previously proposed in the literature. The purpose of this section is to provide a brief quantitative comparison of the proposed method with other reported polyp segmentation methods. However, such a comparison is not a simple task. This is because the implementation details for the reported in literature methods are often not provided. This may include details of the methods’ design parameters or the network training arrangements. Therefore, it is difficult to reproduce evaluation results or make meaningful comparisons with the reported results. Furthermore, the training and test data used by different methods are not always the same. This makes it difficult to compare their performances under the same test conditions. Finally, when reporting on the methods’ performances, some of authors use the test data, which is the same as or highly correlated with the training data. This could happen, for example, because data are split between training/validation and test sets with random image selection rather than random video selection. In the former case it is likely that similar images, taken from a slightly different camera position, of the same polyp, can be separated and assigned to the training and the testing sets. Therefore, such evaluation results may not reflect the real performances of these methods.

The comparison provided here only includes polyp segmentation methods for which Dice coefficient evaluation results have been published (i.e., the averaged Dice coefficient is selected as a measure for methods comparison). The proposed network, as described in this paper, was applied to the segmentation of the CVC-ClinicDB dataset, with a resulting average Dice coefficient of 0.8293.

Table 4 lists results from a sample of other methods reported in literature. Only deep learning-based approaches have been included as they outperform the handcrafted feature-based methods by some margin [24,59]. The shaded cells represent cases where the training and test data overlap and therefore may not reflect a true performance of that method. Some of the methods reported in the table provide results for different configurations. In these cases, the best performing configuration as measured by the average Dice coefficient is indicated in bold; subsequently, these configurations can be used in a direct comparison with the proposed method. It should be made clear that Table 4 does not provide a comprehensive summary of the results reported in the literature, as only the papers using the CVC datasets (which are also used in this paper) and reporting Dice coefficient results have been included. Furthermore, only the methods published in the last two years, which clearly describe the experimental arrangement for selection of the training and test data subsets, have been listed. Interested readers can find more about other recently proposed methods in [60,61,62]. The information about other related colonoscopy image analysis problems, use of handcrafted features, and use of different modalities can be found in [9,24,27,63,64].

From the results in Table 4, it can be seen that the multiple encoder-decoder network (MEDN) [68] and the U-Net with dilatation convolution methods [69] have a comparable performance to the method described in this paper. However, it should be noted that for the reported results the Dilated ResFCN method used only 355 images for training, whereas in [68] 612 images were used. Furthermore, the mean Dice coefficient results reported here for the Dilated ResFCN used 612 test images, whereas results reported in [68] are based on only 196 test images. Although the same test data were used in [69] as in this paper, compared to the results reported here, an additional 10,025 images from the CVC-Clinic VideoDB were used to train the U-Net with the dilatation convolution method. The results reported in [70] are better than for any other method listed in Table 4. However, it seems these results were computed based on a random selection of images into the training and test subsets, rather than a random selection of video sequences, making the interpretation of the method performance somewhat difficult.

The Dilated ResFCN, ResNet-50 FCN8s, and Mask-RCNN were all created based on the ResNet network’s backbone for the deep feature extraction. It can be seen from the results that although the Mask-RCNN: ResNet-101 has a deeper architecture than ResNet-50, its segmentation performance is worse. This is consistent with the experimental results reported in this paper (Section 3), and this further confirms the rationale behind the selection of the ResNet-50 as the base for the feature extraction subnetwork for the proposed Dilated ResFCN network. 

As mentioned above, direct comparison of the methods is rather difficult due to different testing regimes used by different authors. One possible way to overcome this limitation is to evaluate different methods through common challenges. In such cases, all the methods are trained on the same training set and by definition evaluated on the common test subsets using the same set of metrics. Furthermore, as the evaluation is performed at the same time, it can provide a useful snapshot of the current state-of-the-art and reference for further development. The Endoscopic Vision Gastrointestinal Image Analysis (GIANA) polyp segmentation challenges [51] organized at the Medical Image Computing and Computer Assisted Intervention (MICCAI) conferences were designed to provide such snapshots of the state-of-the-art. The Dilated ResFCN network evaluated in this paper was instrumental in securing the first place for the image segmentation tasks at the 2017 GIANA challenge and second place for the SD images at the 2018 GIANA challenge.

## 6. Conclusions

This paper describes Dilated ResFCN, a deep fully convolutional neural network, designed specifically for segmentation of polyps in colonoscopy images. The main objective has been to propose a suitable validation framework and evaluate the performance of that network. The Dilated ResFCN method has been compared against two benchmark methods: FCN8s and ResFCN. It has been shown that suitably selected dilation kernels can significantly improve the performance of polyp segmentation on multiple evaluation metrics. More specifically, it has been shown that the Dilated ResFCN method is the best ay locating polyps with the highest values of the Dice coefficient. It is also good at matching the shape of the polyp with the smallest and most consisted value of the Hausdorff distance. Furthermore, it does not seem to be biased with respect of under/over-segmentation, or indeed polyp shape. However, it preforms better for medium and large polyps. 

The improvement of the method performance has been achieved through test-time augmentation, though that improvement needs to be measured against increased computational cost important for real-time method applications. Further improvement is still required, possibly through additional optimization of the dilation spatial pooling and more effective use of extracted image features, which could potentially be achieved using the squeeze and excitation module [72] recently proposed in literature. Due to a small number of training images, data augmentation is the key for improving segmentation results. It has been shown that in this case rotation is the strongest augmentation technique, followed by local image deformation and color jitter. Overall, the combination of different augmentation techniques has a significant effect on the results. It has been shown that further improvements can be achieved with test time augmentation. The method provides competitive results when compared to other methods reported in the literature. In particular, the method was tested against state-of-the-art at the MICCAI’s Endoscopic Vision GIANA Challenges, securing the first place for the SD and HD image segmentation tasks at the 2017 challenge and second place for the SD images at the 2018 challenge.

## Figures and Tables

**Table 1:** The Dice coefficient results obtained on V1–V4 validation folds for the FCN8s, ResFCN, and Dilated ResFCN. The Dice coefficient and standard deviation, averaged over all validation sets, are also shown.

Network	V1	V2	V3	V4	Average	Standard Deviation
FCN8s	0.68	0.60	0.50	0.75	0.63	0.11
ResFCN	0.68	0.71	0.63	0.82	0.71	0.08
Dilated ResFCN	**0.77**	**0.80**	**0.70**	**0.88**	**0.79**	0.08

**Table 2:** Median and mean values recorded for different measures and segmentation methods.

	Dice		Precision		Recall		Hausdorff	
	Median	Mean	Median	Mean	Median	Mean	Median	Mean
FCN8s	0.69	0.63	0.75	0.68	0.71	0.65	207	193
ResFCN	0.82	0.71	0.89	0.75	0.84	0.74	229	201
Dilated ResFCN	**0.89**	**0.79**	**0.95**	**0.81**	**0.89**	**0.81**	**20**	**54**

**Table 3:** Mean processing time of a single image, without test-time augmentation, using CPU and GPU.

Network	I7-3820 (CPU)	GTX-1080 (GPU)
FCN8s	8.00 s	0.047 c
ResFCN	0.70 s	0.040 s
Dilated ResFCN	1.80 s	0.050 s

**Table 4:** The comparison of existing polyp segmentation methods.

Methods		Dice Coefficient	Training Data	Testing Data
FCN8s [65]		0.810	CVC-ColonDB	
ResNet-50 FCN8s [66]		0.691	CVC-ClinicDB	CVC-ColonDB
		0.323	CVC-ClinicDB	ETIS-Larib
	Resized test images	0.462	CVC-ClinicDB	ETIS-Larib
		0.585	CVC-ColonDB	CVC-ClinicDB
	Pre-processing	0.679	CVC-ClinicDB	CVC-ColonDB
Mask-RCNN [67]	ResNet50	0.716	CVC-ColonDB	CVC-ClinicDB
	ResNet50	0.804	CVC-ColonDB ETIS-Larib	
	ResNet101	0.704	CVC-ColonDB	
	ResNet101	0.775	CVC-ColonDB ETIS-Larib	
Multiple Encoder-Decoder network [68]		0.889	CVC-ClinicDB	
		0.829	CVC-ClinicDB	ETIS-Larib
U-Net with Dilation Convolution [69]		0.825	CVC-ClinicVideoDB CVC-ColonDB GIANA HD	CVC-ClinicDB
Detailed Upsamling Encoder-Decoder Networks [70]		0.913	CVC-ClinicDB	
ResUNet++ [71]		0.7955	CVC-ClinicDB	
